# Patient with a novel syndrome with multiple benign hepatic lesions and extrahepatic neoplasms

**DOI:** 10.1007/s12328-023-01899-3

**Published:** 2023-12-22

**Authors:** Silvana Wilken, Tharusan Thevathasan, Can Kamali, Adrien Guillot, Jana Ihlow, Uli Fehrenbach, Magdalena Danyel, Johann Pratschke, Frank Tacke, Felix Krenzien

**Affiliations:** 1 Department of Surgery, Campus Charité Mitte and Campus Virchow-Klinikum, Charité – Universitätsmedizin Berlin, Corporate Member of Freie Universität Berlin and Humboldt-Universität Zu Berlin, Charitéplatz 1, 10117 Berlin, Germany; 2 Department of Cardiology, Campus Benjamin Franklin, Charité - Universitätsmedizin Berlin, Corporate Member of Freie Universität Berlin and Humboldt-Universität Zu Berlin, Hindenburgdamm 30, 12203 Berlin, Germany; 3 Berlin Institute of Health at Charité – Universitätsmedizin Berlin, BIH Biomedical Innovation Academy, Charitéplatz 1, 10117 Berlin, Germany; 4 Department of Hepatology and Gastroenterology, Campus Virchow-Klinikum and Campus Charité Mitte, Charité Universitätsmedizin Berlin, Corporate Member of Freie Universität Berlin and Humboldt-Universität Zu Berlin, Charitéplatz 1, 10117 Berlin, Germany; 5 Institute of Pathology, Charité-Universitätsmedizin Berlin, Corporate Member of Freie Universität Berlin and Humboldt-Universität Zu Berlin, Charitéplatz 1, 10117 Berlin, Germany; 6 Clinic for Radiology, Charité - Universitätsmedizin Berlin, Corporate Member of Freie Universität Berlin and Humboldt-Universität Zu Berlin, Charitéplatz 1, 10117 Berlin, Germany; 7 Institute of Medical Genetics and Human Genetics, Charité - Universitätsmedizin Berlin, Corporate Member of Freie Universität Berlin, Humboldt-Universität Zu Berlin, and Berlin Institute of Health, 13353 Berlin, Germany; 8 Department of Surgery, Campus Charité Mitte and Campus Virchow-Klinikum, Charité- Universitätsmedizin Berlin, Augustenburger Platz 1, 13353 Berlin, Germany

**Keywords:** Benign liver tumor, Hepatocellular adenoma, Focal nodular hyperplasia, Hepatic hemangioma

## Abstract

Simultaneous occurrence of benign hepatic lesions of different types is a sporadic phenomenon. To the best of our knowledge, we report the first clinical case of a syndrome with simultaneous manifestations of three different entities of benign liver tumors (hepatocellular adenoma, focal nodular hyperplasia and hemangioma) with a novel mutation detected in the liver adenoma and in the presence of a number of further extrahepatic organ neoplasms. Furthermore, we describe for the first time the presence of liver epithelial cells of hepatocytic phenotype expressing cytokeratin 7 (CK7) at the border of the adenoma. These findings may be important for explaining pathogenesis of benign as well as malignant tumors based on genetic and histopathological features.

## Introduction

Benign liver tumors are a heterogeneous group of lesions that are mostly found incidentally in clinical examinations. Depending on the subtype, benign lesions may require surgical treatment in cases of clinical symptoms, risk of bleeding or excessive size progression [1]. Multiple risk factors for liver tumorigenesis have been discovered for certain entities, such as female sex, genetic mutations, such as in HNF1A or CTNNB1, or exogenous estrogen intake [2–4]. Simultaneous occurrence of benign liver tumors of different types is a sporadic phenomenon and has mostly been discussed as a coincidental event rather than a syndrome [5–9].

Herein we report the clinical case of a female patient with simultaneous manifestation of hepatocellular adenoma (HCA), giant hepatic hemangioma (HH) and focal nodular hyperplasia (FNH).

## Case presentation

Almost two decades after the initial detection of a liver lesion, a 58-year-old female Caucasian patient with concomitant manifestations of HCA, giant HH and FNH was admitted in 2020 to the Department of Surgery for further diagnostic and therapeutic evaluation. The patient denied symptoms indicative for liver tumors, despite a recent history of hepatomegaly with mild perihepatic and peripancreatic ascites, significantly progressing liver lesions with compression of intrahepatic bile ducts and gallbladder. Alpha fetoprotein (AFP) and carcinoembryonic antigen (CEA) levels were normal, while transaminase, gamma-glutamyltransferase, alkaline phosphatase and C-reactive protein levels had been elevated progressively since 2003, reaching values of over three times the upper bound of the normal range of these parameters.

### Patient history

**Fig. 1:**
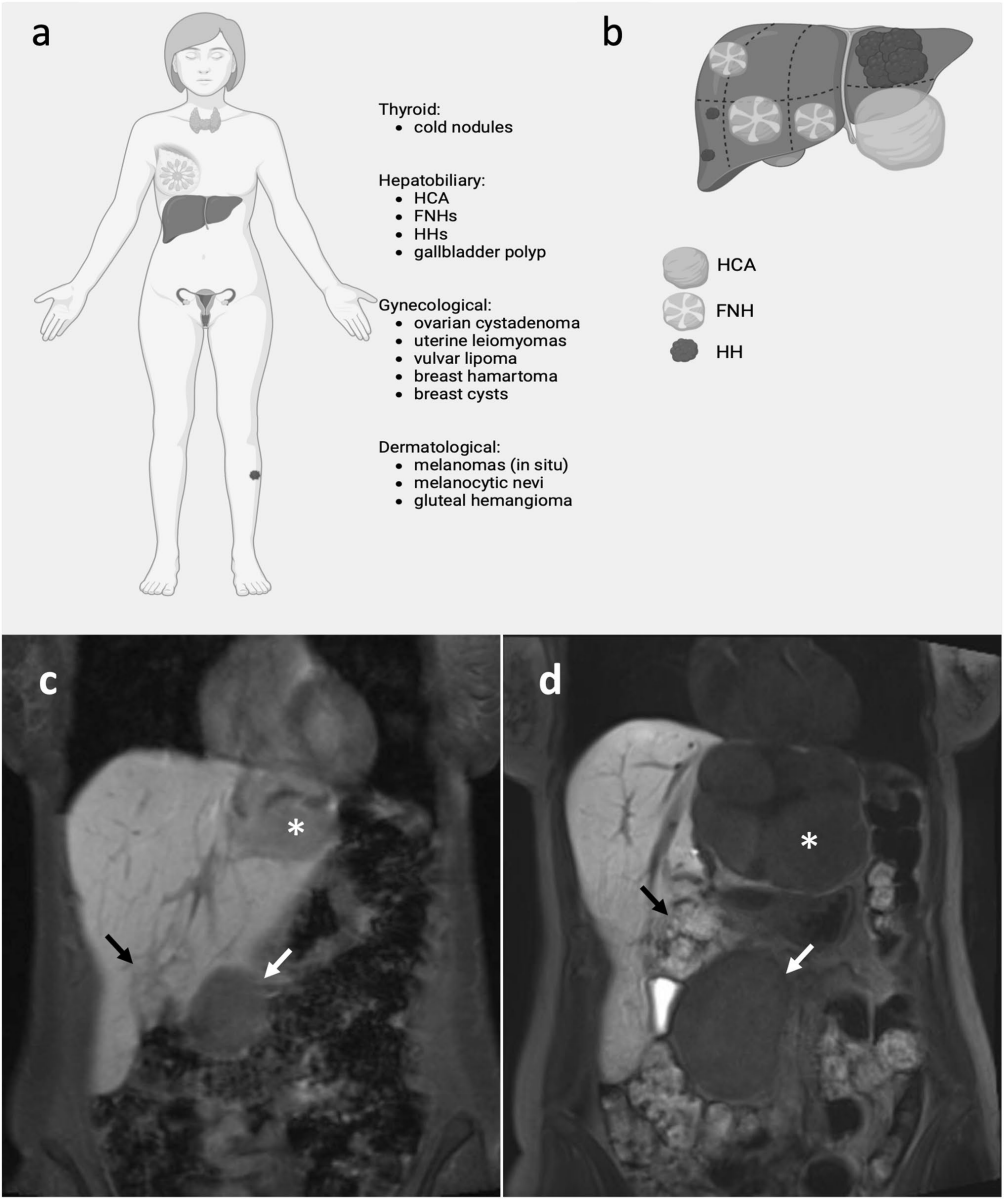
**a**, **b** Schematic presentation of all the neoplasms of the index patient (**a**) and of the benign liver lesions (**b**), comprising an exophytic hepatocellular adenoma (HCA) in segment 3, focal nodular hyperplasia (FNH) in segments 4b, 5 and 7, giant confluent hepatic hemangioma (HH) in segment 2, as well as smaller HHs in segment 6. This figure was created with BioRender.com. **c**, **d** Abdominal MRI, T1 sequence in coronal plane: **c** in 2003 and **d** in 2020 with gadoxetic acid showing a hepatocellular adenoma (HCA) in segment 3 (white arrow) presenting features of HNF1A-mutated HCA, a focal nodular hyperplasia (FNH) (black arrow) in segment 4b and a giant confluent hepatic hemangioma (HH) in segment 2 (white asterisk)

The patient presented numerous multi-organ neoplasms over the time span of two decades, including hepatic (HCA, FNH and HH), gynecologic (ovarian serous cystadenoma, uterine leiomyomas, calcified breast hamartoma and cysts, vulvar lipoma), endocrine (thyroid nodules), dermatologic (multiple melanocytic nevi and melanomas in atypical regions, gluteal hemangioma) and biliary (gallbladder polyp) (Fig. 1a, b). A liver lesion was first detected in the patient incidentally during a laparoscopic oophorectomy in 2002. Pathologic examinations, ultrasound and native magnetic resonance imaging (MRI) revealed a suspicious lesion, consistent with either FNH or HCA, without certain differentiation available at that time. The clinicians’ recommendation was clinical surveillance with regular follow-up imaging. Sensitive contrast-enhanced MRI (CE-MRI) with gadoxetic acid in 2014 yielded the definitive diagnosis of a single HCA (diameter 90 mm) and multiple HH and FNH distributed throughout almost all liver segments. Esophagogastroduodenoscopy and colonoscopy were inconspicuous. Other pathological findings included mild hepatic steatosis, self-limiting hepatitis E infection, gallbladder concrements, urethral prolapse and a subphrenic cyst. By the time of diagnosis of the first liver lesion in 2002, the patient had been taking oral contraceptives for roughly 15 years. Family history was positive for the occurrence of neoplasms, as the mother of the patient had a benign liver lesion (entity unknown but consistent with either HCA, FNH or HH) and a leiomyoma.

### Diagnostic and therapeutic management

We confirmed the diagnosis of benign liver tumors with CE-MRI (Fig. 1c, d). In accordance with the guidelines of The European Association for the Study of the Liver [1]*,* we subjected the patient to laparoscopic left lateral sectionectomy with the additional removal of segment 4a. The therapeutic decision was based on multidisciplinary team consensus due to new abdominal complaints, increased transaminases and tumor size (diameter 90 mm). The surgical specimen confirmed as expected HCA and multiple HHs in the resected liver segments.

### Histology

**Fig. 2:**
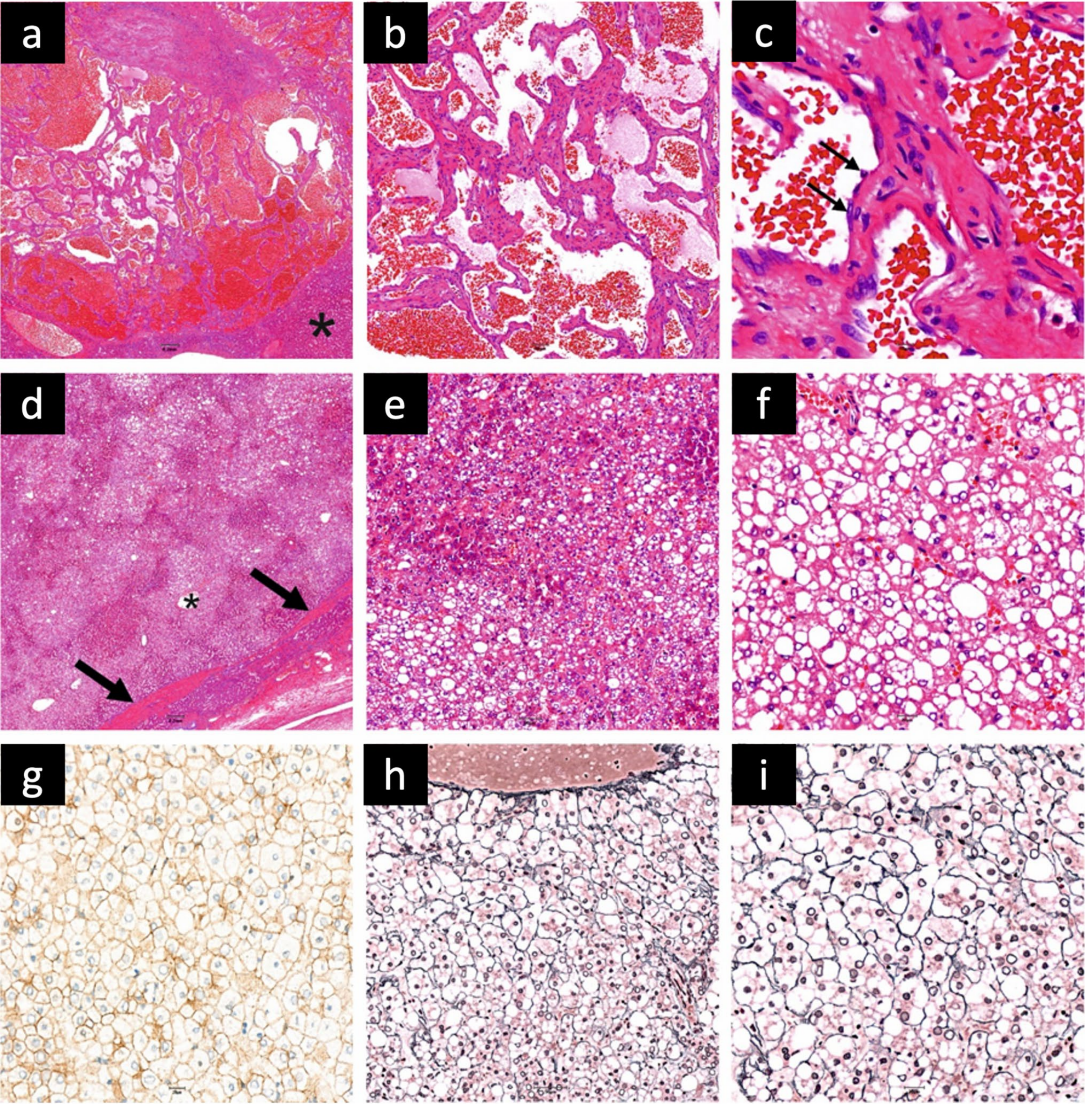
Histopathology and immunohistochemistry of liver findings in a female 58-year-old patient. **a–c** Intrahepatic cavernous hemangiomas surrounded by residual liver parenchyma (*) and lined by a single layer of bland endothelial cells without mitoses (thin arrows). **d–i** Hepatocellular adenoma, not otherwise specified. **d** The adenoma is arranged in a pseudoacinar pattern and shows a well-defined border (thick arrows), singular small arteries (*) and steatotic foci. **e**, **f** The nuclei contain glycogen bodies, but no atypia. **g** Beta-catenin is not expressed in the nuclei. **h–i** Gomori stain reveals an intact reticulin network that surrounds predominantly thin hepatocytic cell plates with a thickness of 1–2 cells. Resolution: **a**, **d**: 2 × 10^5^ / **b**, **e**, **h**: 5 × 10^7^ / **f**, **g**, **i**: 2 × 10.^7^

We performed postoperative histopathological, immunohistochemical and molecular analyses of the HCA and surrounding liver tissue. Tissue sections had a thickness of 4 µm and were stained using hematoxylin and eosin stain (H&E), periodic acid–Schiff stain, Gomori silver stain as well as immunohistochemical stains for β-catenin (Carboxy-terminal Antigen, polyclonal, Cell Signaling, 1:50) and serum amyloid (SAA, clone mc1, Dako, 1:600). Histopathology revealed a capsuled lesion congruous with an HNF1A-inactivated HCA with diffuse fat accumulation in the parenchyma. The hepatocytes in this lesion partially appeared to be ballooned and in a pseudoglandular configuration (a summary of the histopathological and immunohistochemical findings in the adenoma are presented in Fig. 2). At the edge area of the lesion, these cells presented a weak expression of serum amyloid A (SAA), which, however, was not elevated compared to the adjacent healthy liver tissue.

**Fig. 3:**
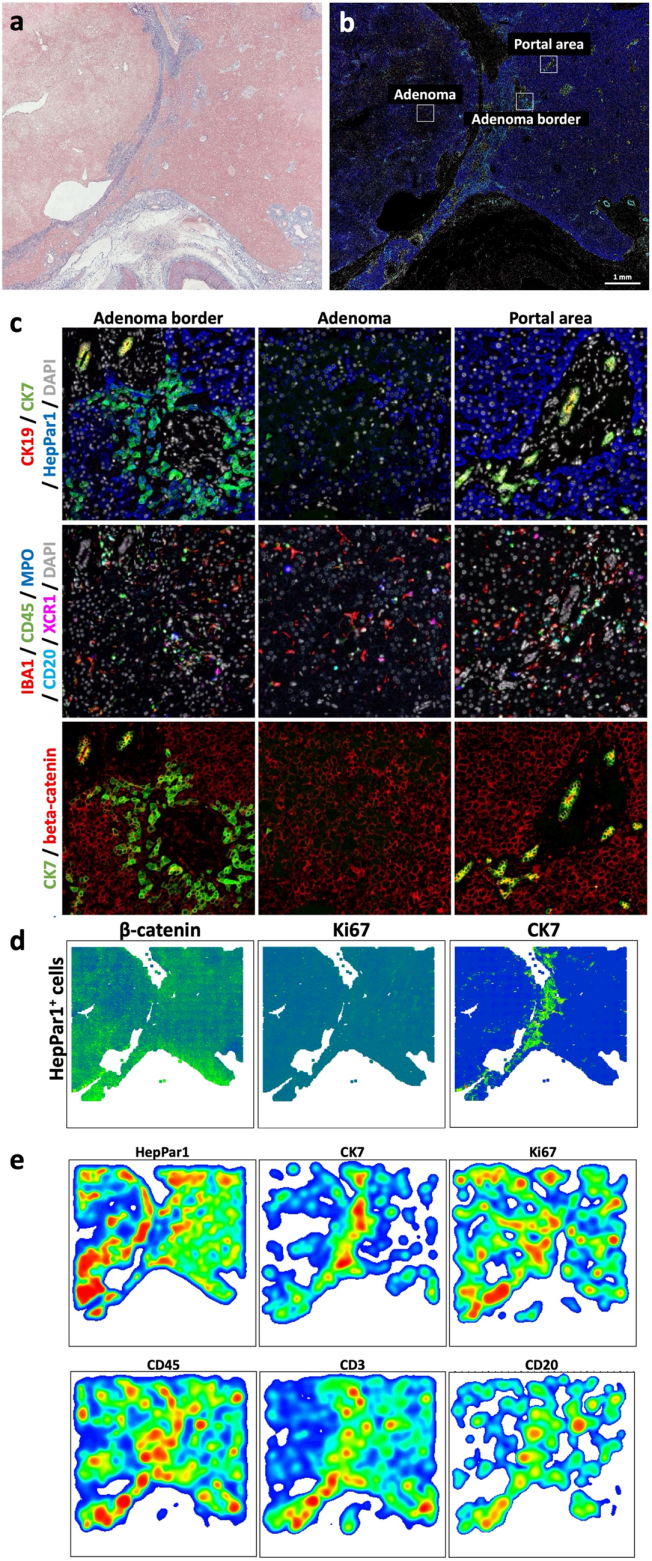
Sequential immunostaining was performed as previously described [10] on a liver section containing healthy liver tissue, adenoma and adenoma border. Representative pictures of **a** Masson’s trichrome staining and **b** multiplex immunostaining. **c** Higher magnifications corresponding to the areas marked in (**b**). **d** Single cell plots representing the localization of HepPar1-positive cells. Dot colors indicate the staining intensities for beta-catenin, Ki67 or CK7, as indicated (green = high intensity, blue = low intensity). **e** Heatmaps showing cellular distribution and density of cells expressing HepPar1, CK7, Ki67, CD45, CD3 and CD20 (red = high density, blue = low density)

We performed further sequential multiplex immunostaining as previously described [10] to explore the expression of specific cell type markers and mitotic activity throughout the HCA and healthy liver tissue (a selection of the most important findings of the multiplex immunostaining is presented in Fig. 3). The mitotic activity in the adenoma measured by Ki67 was not different from the rest of the tissue. The adenoma cells showed consistent HepPar1 expression, confirming their hepatocytic origin [11, 12]. Remarkably, along the adenoma border, a concentration of cells expressing CK7 and HepPar1 was observed. Interestingly, these cells presented the morphology of hepatocytes and not of ductular cells.

### Focused next-generation sequencing

Focused next-generation sequencing (oncomine focus assays) of the HCA tissue revealed a novel missense variant in the hepatocyte nuclear factor-1 alpha gene (gene name: *HNF1A*, NM_000545: Exon 3–4)): c.613A > G, p.K205E with 37% allele frequency (i.e., heterozygous). Interestingly, the *HNF1A* gene in healthy liver tissue was wild type, suggesting a possible association of the above-mentioned mutation with tumorigenesis in the context of a somatic and not a germline mutation. Other pathological characteristics of the adenoma tissue as well as MRI imaging were highly suggestive of HNF1A-inactivated HCA.

### Exome sequencing

We also performed exome sequencing of the patient’s DNA (extracted from a blood sample) to examine for germline mutations that could be the cause of the patient’s phenotype. The clinical features of the patient were addressed using the Human Phenotype Ontology [13]. The following terms were used for the subsequent analysis: hepatocellular adenoma (HPO: 0012028), hemangioma (HPO: 0001028), melanoma (HPO: 0002861) and lipoma (HPO: 0012032). No disease-causing mutation could be identified (supplementary material on the exome sequencing analysis can be acquired upon request from the corresponding author). In particular, there were no pathogenic germline variants in *HNF1A*. The comparison of the patient exome with the known mutations associated with the above-mentioned phenotypes showed no match with no evidence for a monogenic cause.

## Discussion

Our case report describes a syndrome-like synchronous manifestation of HH, HCA and FNH in the liver, as well as numerous, mostly benign tumors in multiple organs. To the best of our knowledge, the concomitant manifestation of HH, HCA and FNH has been reported only once in 2003 [5]. In this case, the authors brought up the hypothesis that these three benign lesions might share a common origin, a vascular anomaly, in particular. In our case, we further performed whole exome sequencing, advanced molecular pathology and fluorescence microscopy to achieve better understanding of the pathological pattern of the patient.

The sequence analysis of the *HNF1A* gene revealed a novel heterozygous variant (c.613A > G; p.(K205E)) in the HCA and wild type in the healthy liver tissue. To the best of our knowledge, this genetic variant has neither been documented in any database nor has it been mentioned in the literature regarding liver lesions or any other neoplasms. Thus, the clinical significance of this variant is unknown and yet to be clarified in regard to the occurrence of benign liver lesions in the presence of multiple extrahepatic neoplasms. Remarkably, *HNF1A* gene mutations defining the HNF1A-inactivated HCA are biallelic [14]; however in our case, no second variant could be identified.

The immunofluorescence analysis uncovered CK7-positive liver epithelial cells at the adenoma border. The phenomenon of CK7^+^HepPar1^+^ cells has been observed in various non-neoplastic liver diseases in humans, such as viral hepatitis, autoimmune hepatitis and primary sclerosing cholangitis [15, 16], as well as in hepatocellular carcinoma [17]. These cells have also been reported to be present throughout the tumor mass of some HCAs as well as in FNH, in which cases the intermediate phase between progenitor cells and differentiated hepatocytes or cholangiocytes was presumed [18, 19]. Notably, in the current case, CK7^+^HepPar1^+^ cells were not observed in the tumor itself, but at the border of healthy liver tissue to the HCA. This could be as a result of local cholestasis in the surrounding healthy liver tissue induced by the HCA and its substantial size, also explaining the elevated cholestatic parameters of the patient. Alternatively, the CK7^+^HepPar1^+^ cells could represent hepatic progenitor cells and indicate a regenerative response [20]. Further investigation is needed to examine if this is a common phenomenon in HCA or an observation specific for this case of multiple benign liver lesions.

Here, we present a novel unknown syndrome described by many multiple benign neoplasms in the liver and other glands. This finding may be important for explaining the pathogenesis of benign as well as malignant tumors based on genetic and histopathological findings.
